# Association of physical function with predialysis blood pressure in patients on hemodialysis

**DOI:** 10.1186/1471-2369-15-177

**Published:** 2014-11-15

**Authors:** Adrian P Abreo, David Glidden, Patricia Painter, Janice Lea, Charles A Herzog, Nancy G Kutner, Kirsten L Johansen

**Affiliations:** Division of Nephrology, University of Cincinnati College of Medicine, 231 Albert Sabin Way, MSB,G261, Cincinnati, OH 45267 USA; Department of Epidemiology & Biostatistics, University of California, San Francisco, CA USA; Department of Physical Therapy, University of Utah, Salt Lake City, UT USA; Division of Nephrology, Emory University, Atlanta, GA USA; Division of Cardiology, Hennepin County Medical Center and University of Minnesota, Minneapolis, MN USA; United States Renal Data System, Cardiovascular Special Studies Center, Minneapolis, MN USA; United States Renal Data System Rehabilitation/Quality of Life Special Studies Center, Atlanta, USA; Nephrology Section, San Francisco VA Medical Center, 111J, 4150 Clement Street, San Francisco, CA 94121 USA; United States Renal Data System Nutrition Special Studies Center, San Francisco, USA; Division of Nephrology, University of California, San Francisco, CA USA; Nephrology Section, 111J, 4150 Clement Street, 94121 San Francisco, CA USA

**Keywords:** Blood pressure, End-stage renal disease, Physical function

## Abstract

**Background:**

New information from various clinical settings suggests that tight blood pressure control may not reduce mortality and may be associated with more side effects.

**Methods:**

We performed cross-sectional multivariable ordered logistic regression to examine the association between predialysis blood pressure and the short physical performance battery (SPPB) in a cohort of 749 prevalent hemodialysis patients in the San Francisco and Atlanta areas recruited from July 2009 to August 2011 to study the relationship between systolic blood pressure and objective measures of physical function. Mean blood pressure for three hemodialysis sessions was analyzed in the following categories: <110 mmHg, 110-129 mmHg (reference), 130-159 mmHg, and ≥160 mmHg. SPPB includes three components: timed repeated chair stands, timed 15-ft walk, and balance tests. SPPB was categorized into ordinal groups (≤6, 7-9, 10-12) based on prior literature.

**Results:**

Patients with blood pressure 130-159 mmHg had lower odds (OR 0.57, 95% CI 0.35-0.93) of scoring in a lower SPPB category than those whose blood pressure was between 110 and 129 mmHg, while those with blood pressure ≥160 mmHg had 0.56 times odds (95% CI 0.33-0.94) of scoring in a lower category when compared with blood pressure 110-129 mmHg. When individual components were examined, blood pressure was significantly associated with chair stand (130-159 mmHg: OR 0.59, 95% CI 0.38-0.92) and gait speed (≥160 mmHg: OR 0.59, 95% CI 0.35-0.98). Blood pressure ≥160 mmHg was not associated with substantially higher SPPB score compared with 130-159 mmHg.

**Conclusions:**

Patients with systolic blood pressure at or above 130 mmHg had better physical performance than patients with lower blood pressure in the normotensive range. The risk-benefit tradeoff of aggressive blood pressure control, particularly in low-functioning patients, should be reexamined.

## Background

Recent studies have suggested the need for further examination of the relationship between blood pressure control and physical function. The Action to Control Cardiovascular Risk in Diabetes (ACCORD) trial showed more adverse events among patients with diabetes randomly assigned to a systolic blood pressure target of <120 mmHg compared with a target of <140 mmHg without reduction in cardiovascular outcomes [1]. Despite positive results of some randomized controlled trials of intensive blood pressure control [2], several randomized studies have also called into question the benefit of aggressive blood pressure control among elderly or frail individuals [3]. A recent study found that the association of high blood pressure and mortality varied by walking speed in elderly patients such that elevated blood pressure was not associated with higher mortality among patients with slow gait speed [4]. Taken together, these findings raise the possibility that strict blood pressure control could contribute to poor function among elderly or frail individuals by increasing symptoms of dizziness and fatigue and lowering physical activity and may increase the risk of adverse outcomes without improving outcomes. Indeed, updated practice guidelines for the management of high blood pressure in adults have raised the recommended systolic blood pressure treatment goal to <150 mmHg for individuals aged 60 years or older, citing evidence of a lack of benefit of treatment to lower targets [5].

Patients with poor physical function could be at higher risk for these complications and simultaneously be less likely to benefit. These issues are particularly relevant among patients on hemodialysis because approximately seventy-six percent of dialysis patients are prescribed antihypertensive medications, and they experience significant impairments in physical function that have been associated with higher mortality in chronic kidney disease and community-dwelling elderly populations [6–9]. Although studies have shown that rigorous exercise interventions can improve physical function among patients with ESRD, these interventions are resource-intensive and not accessible to all patients [10, 11]. Thus, it is important to consider other factors that might affect physical function in this population.

We used data from the United States Renal Data System (USRDS) ACTIVE/ADIPOSE (A Cohort study To Investigate the Value of Exercise/Analyses Designed to Investigate the Paradox of Obesity and Survival in ESRD) cohort, which includes measurements that make up the short physical performance battery (SPPB), to study the relationship between levels of blood pressure and objective measures of physical function in hemodialysis patients. We hypothesized that lower blood pressure would be associated with worse performance on the SPPB. In addition to examining blood pressure that was frankly low, we were also interested in examining whether blood pressure in ranges considered to indicate good blood pressure control would be associated with worse performance.

## Methods

The ACTIVE/ADIPOSE is a cohort study of the United States Renal Data System (USRDS) Nutrition and Rehabilitation/Quality of Life Special Studies Centers that enrolled 778 prevalent hemodialysis patients from the San Francisco Bay Area and the Atlanta metropolitan area between 2009 and 2011. The description of the ACTIVE/ADIPOSE study and methods have been published elsewhere [12]. English or Spanish-speaking patients who had been on dialysis for at least 3 months were included. Study participants provided written informed consent, and the study was approved by the Institutional Review Boards at Emory University and the University of California, San Francisco. The study was conducted in adherence with the Declaration of Helsinki.

We performed a cross-sectional analysis to examine the association between predialysis systolic blood pressure and components of the short physical performance battery among 749 patients who had blood pressure and physical function data available.

### Short physical performance battery (SPPB)

Our primary outcome, the SPPB was designed to assess lower extremity function in community-dwelling elderly individuals and includes three components: (1) timed repeated chair stands, (2) a timed 15-foot walk, and (3) balance testing over ten seconds. We performed SPPB assessments as described by Guralnik *et al*. prior to a mid-week or end-of-week dialysis session [13].

Participants were asked to stand up from a chair and sit down again five times repeatedly as quickly as possible. A score of zero was given if a participant was unable to perform five chair stands. A score of one, two, three, or four was assigned to a participant who completed five chair stands in ≥16.7, 13.7-16.6, 11.2-13.6, and ≤11.1 seconds, respectively.

Participants were asked to walk a marked 15-foot course at a normal pace. Two trials were conducted, and the faster of the two walks was used for analysis. Gait speed was calculated for each patient using distance in meters and time in seconds. Patients were scored from 0 to 4, where 0 points indicated inability to perform the walk and 4 indicated a gait speed of >0.83 m/s.

Balance testing was comprised of three components: (1) semitandem stand (2) side-by-side stand and (3) tandem stand. Participants began with a semi-tandem stand where the heel of one foot was placed to the side of the first toe of the other foot. Those who were able to stand for 10 seconds in the semi-tandem stand position were tested in the full tandem stand with the heel of one foot directly in front of the toes of the other. Balance testing was scored on a 0-4 scale with a score of 4 indicating a full tandem stand for 10 seconds. Total SPPB score was calculated by the sum of the components.

### Blood pressure and antihypertensive medication

Although postdialysis blood pressure and 24-hour ambulatory blood pressure monitoring may be important predictors of outcomes in the hemodialysis population [14, 15], we chose predialysis systolic blood pressure as our primary predictor in this analysis because the SPPB was measured prior to a dialysis session. Predialysis blood pressures taken as part of routine care were recorded for the previous three hemodialysis sessions before the study visit, and the mean systolic blood pressure was used as the primary predictor. We analyzed predialysis blood pressure in the following categories: <110 mmHg, 110-129 mmHg, 130-159 mmHg, and ≥160 mmHg. We chose these categories to be similar to other studies examining outcomes related to blood pressure among patients on hemodialysis. For example, patients with predialysis blood pressure less than 110 mmHg had a higher risk of death in a US-based observational study [16]. A study using international data from the Dialysis Outcomes and Practice Patterns Study concluded that survival was significantly better among patients with predialysis SBP ≥130 mmHg and at facilities with more patients at predialysis SBP 130 to <160 mmHg [17]. We separated patients with a blood pressure of <110 mmHg from those with a blood pressure of 110-129 mmHg to ensure that the results were not driven by patients with very low blood pressure who had poor physical function. Information about prescription medication was carefully collected through chart review, and the number of prescribed antihypertensive medications was quantified for each participant.

### Covariates

Data on comorbidities were obtained from the information recorded on the Medical Evidence Form 2728 and included in the USRDS. Comorbidities from the Medical Evidence Form 2728 have been previously validated with sensitivity being high for hypertension and diabetes and intermediate for peripheral vascular disease, cerebrovascular disease and heart failure [18]. Serum albumin was measured by nephelometry. Covariates were selected based on a clinical conceptual model and included age, gender, African-American race, diabetes mellitus, heart failure, coronary artery disease, stroke, peripheral vascular disease, ESRD vintage (date of first ESRD hemodialysis treatment), hemoglobin, body mass index, interdialytic weight gain, and serum albumin concentrations. Interdialytic weight gain was calculated by subtracting the postdialysis weight from the predialysis weight of the subsequent treatment and dividing by the postdialysis weight. The most recent clinical hemoglobin level documented in the chart was used for the analysis.

### Statistical analysis

We compared patient characteristics using chi squared tests, t-tests, Mann-Whitney U tests, and linear regression as appropriate. Restricted cubic splines were generated to examine the association between total SPPB score and mean predialysis blood pressure.

We used univariate and multivariate ordinal logistic regression with predialysis blood pressure categories as the primary predictor and predialysis SBP of 110-129 mmHg as the reference category. Models were constructed with the SPPB and each of the component measures as outcomes. Total SPPB score was categorized into three ordinal groups (≤6, 7-9, 10-12) as performed in prior studies [19]. We examined all residuals of continuous variables for a normal distribution and those with non-normal distribution (ESRD vintage) were log-transformed. Missing serum albumin values (n = 15, 2%) were accounted for using multiple imputations. The multivariate model was run with and without data on prescribed antihypertensives. Multiple binary cutoffs were examined to ensure that the data met the proportional odds assumption. Because medications prescribed to treat heart failure or coronary heart disease can lower blood pressure, we performed sensitivity analyses excluding patients with each of these diagnoses. All analyses were completed using Stata 13 (StataCorp LP).

## Results

The mean age of the cohort was 57.3 years (SD 14.2). Patients in the highest blood pressure category were more likely to be African American and have higher interdialytic weight gains, while those in the lowest category had lower serum albumin (3.8 ± 0.7 mg/dl) than those in other groups (p = 0.02). Fifty-six percent of patients in the lowest blood pressure category and 75% of patients in the 110-129 mmHg group were prescribed at least one antihypertensive medication. There were no statistically significant differences in prevalence of comorbidities based on blood pressure (Table 1).

**Table 1:** **Baseline characteristics of participants based on blood pressure category**

Characteristics	Predialysis SBP <110 mmHg	Predialysis SBP 110-129 mmHg	Predialysis SBP 130-159 mmHg	Predialysis SBP ≥160 mmHg	p-value ^┼^
	N = 27	N = 92	N = 373	N = 257	
**Age (years)**	56.2(15.1)	58.9(14.9)	58.2(14.4)	55.5(13.6)	0.07
**Gender**					
**▪Male**	19(70%)	52(57%)	234(63%)	139 (54%)	0.09
**Race**					
**▪White**	5(19%)	31(34%)	103 (28%)	39(15%)	<0.001
**▪African American**	14(52%)	48 (52%)	207(56%)	192(75%)	
**▪Other** ^*^	8(30%)	13(14%)	63(17%)	26(10%)	
**Comorbidities**					
**▪Coronary Artery Disease**	5(19%)	10(11%)	30(8%)	21(8%)	0.26
**▪Diabetes Mellitus**	10(37%)	34(37%)	179(48%)	122(47%)	0.19
**▪Stroke**	1(4%)	5(5%)	15(4%)	12(5%)	0.93
**▪Heart Failure**	7(26%)	18(20%)	75(20%)	42(16%)	0.51
**▪Peripheral Vascular Disease**	4(15%)	12(13%)	34(9%)	22(9%)	0.47
**Laboratory Values**					
**▪Hemoglobin (g/dl)**	11.4(1.3)	11.3(1.3)	11.8(3.1)	11.8(2.4)	0.38
**▪Albumin (g/dl)**	3.8 (0.7)	4.0(0.3)	4.0(0.3)	4.0(0.4)	0.02
**ESRD Vintage (years) [median, (25th, 75th percentile)]**	4.3 (2.1,10.5)	3.2(1.5,6.0)	2.8(1.3,6.0)	3.7(1.7,7.4)	0.08
**Number of antihypertensive prescribed [median, (25th, 75th percentile)]**	1(0,1)	1.5(0.5, 3)	2(1,3)	3 (2,3)	<0.001
**Body Mass Index (kg/m** ^**2**^ **)** ^**ơ**^					
**▪ < 20**	0(0%)	8(9%)	13(4%)	10(4%)	0.004
**▪20-24.99**	3 (11%)	24(26%)	100(27%)	88(34%)	
**▪25-29.99**	6(22%)	32(35%)	109(29%)	75(29%)	
**▪30-34.99**	10(37%)	13(14%)	80(21%)	41(16%)	
**▪ ≥ 35**	1(4%)	1(1%)	1(0.2%)	0(0%)	
**Average IDWG (%)**	2.8(1.8)	3.2(1.8)	3.4(1.9)	3.5(1.9)	0.005

^┼^p-value refers to χ^2^ test (for gender, race, BMI, and comorbities), linear regression for continuous variables (age, hemoglobin, albumin, hemodialysis vintage, antihypertensives).

*Includes 1 missing race value.

^ơ^Includes 1 missing value from each of the following categories: <110, 110-129, 130-159.

IDWG: Interdialytic weight gain, expressed as a percentage of predialysis body weight.

**Figure 1:**
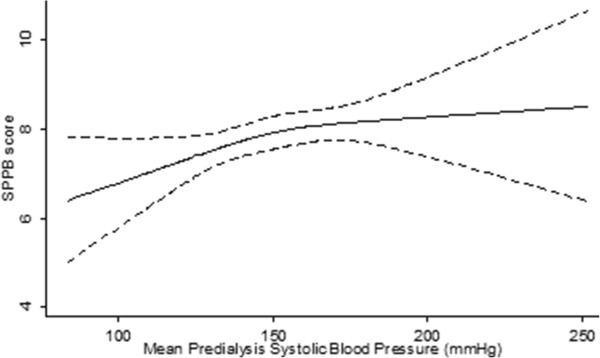
**Spline curve for the unadjusted association between mean predialysis systolic blood pressure and short physical performance battery score (solid line) with 95% confidence intervals (dashed lines).**

A restricted cubic spline curve for the unadjusted associated between mean predialysis blood pressure and SPPB score is displayed in Figure 1. Performance was better at higher blood pressures, but the slope of this association flattened above 151 mmHg. Examining SPPB by category, 29% of participants scored ≤6 points and 45% scored ten or more points on the SPPB. The odds ratio of scoring in a lower SPPB category (0-6) was 0.60 (95% CI 0.39-0.94) for participants with a predialysis mean systolic blood pressure of ≥160 mmHg when compared with the reference range of 110-129 mmHg in univariate analysis. In a multivariate model, participants with blood pressures 130-159 mmHg and ≥160 mmHg had significantly lower odds of scoring in a lower SPPB category (130-159 mmHg: OR 0.57 (95% CI 0.35-0.93), ≥160 mmHg: OR 0.56, (95% CI 0.33-0.94); Table 2). Blood pressure less than 110 mmHg was not associated with worse function than 110-129 mmHg (OR 0.82, 95% CI 0.32-2.09). Diabetes mellitus, older age, peripheral artery disease, stroke history, female gender, and African-American race were associated with worse SPPB scores, and higher serum albumin concentration was associated with higher scores. Neither heart failure nor hemoglobin concentration was significantly associated with physical performance.

**Table 2:** **Odds ratio of scoring in a lower SPPB category**

	OR (95% CI)	p-value
***Mean predialysis systolic BP (mmHg)***		
**<110**	0.82(0.32-2.09)	0.67
**110-129**	Reference	
**130-159**	0.57(0.35-0.93)	0.03
**≥160**	0.56(0.33-0.94)	0.03
***Demographics***		
**Age (per 10 yrs)**	1.83(1.60-2.09)	<0.001
**Male**	0.47(0.34-0.65)	<0.001
**African-American race**	1.77(1.25-2.50)	0.001
***Comorbidity***		
**Stroke**	2.29(0.98-5.35)	0.06
**Diabetes mellitus**	1.95(1.40-2.73)	<0.001
**Serum albumin (per 0.5 g/dl)**	0.55(0.44-0.68)	<0.001
**Peripheral Vascular Disease**	3.77(2.09-6.81)	<0.001
**Hemoglobin**	0.95(0.89-1.02)	0.2
**Heart Failure**	1.44(0.96-2.16)	0.08
**Coronary artery disease**	1.10(0.62-1.98)	0.74
**ESRD vintage (log)**	1.06(0.92-1.23)	0.38
**Average IDWG (%)**	1.05 (0.96-1.15)	0.31
***Body Mass Index***		
**<20**	0.61(0.26-1.43)	0.26
**20-24.99**	Reference	
**25-29.99**	0.89(0.59-1.34)	0.58
**30-34.99**	1.44(0.91-2.28)	0.12
**≥35**	1.52(0.95-2.43)	0.08

IDWG: Interdialytic weight gain as a percentage of predialysis body weight.

The association between higher blood pressure and higher SPPB score remained statistically significant when the number of prescribed antihypertensive medications was added to the model (130-159 mmHg: OR 0.58, 95% CI 0.35-0.95), and there was no statistically significant interaction between systolic blood pressure and number of antihypertensive medications prescribed.

**Figure 2:**
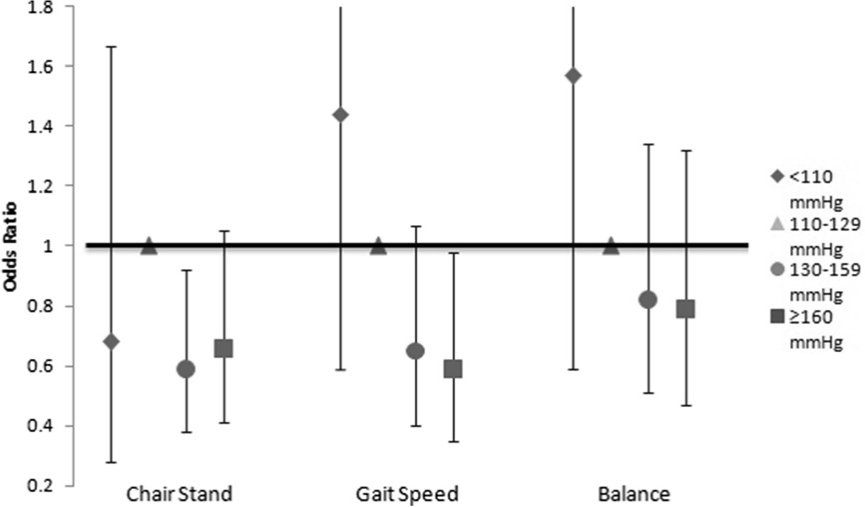
**Odds ratio of lower performance category for each test based on predialysis systolic blood pressure category.**

When we examined the individual components of the SPPB in multivariable analysis, chair stand performance and gait speed were significantly associated with blood pressure while balance was not (Figure 2). The odds ratio of scoring in a lower chair stand category was 0.59 (95% CI 0.38-0.92) in the 130-159 mmHg group and 0.66 (95% CI 0.41-1.05) for the ≥160 mmHg group when compared to the reference group. The odds ratio of scoring in a lower category of walking speed was 0.65 (95% CI 0.40-1.07) for the 130-159 mmHg category and 0.59 (95% CI 0.35-0.98) for the ≥160 mmHg group when compared with the reference group. Results of the multivariate models for chair stand, gait speed, and balance are displayed in Figure 2.

### Sensitivity analyses

Because antihypertensive medications used in the treatment of heart failure or coronary artery disease could influence predialysis blood pressure, we conducted sensitivity analyses excluding participants with these diagnoses. When patients with heart failure (n = 142) were excluded from the primary analysis, the odds of scoring in a lower SPPB category remained similar (130-159 mmHg: OR 0.58, 95% CI 0.33-1.00; ≥160 mmHg: OR 0.57, 95% CI 0.33-1.00). When patients with coronary heart disease were excluded from the analysis (n = 66), the point estimates were also similar (130-159 mmHg: OR 0.61, 95% CI 0.36-1.03; ≥160 mmHg: OR 0.59, 95% CI 0.34-1.02).

## Discussion

We have demonstrated that a predialysis systolic blood pressure of 130 mmHg or more is associated with better physical performance on the SPPB when compared with a predialysis systolic blood pressure of 110-129 mmHg among patients on hemodialysis. The relationship between low blood pressure and physical performance persisted even after adjustment for age and important comorbidities and even when we adjusted for number of prescribed antihypertensive medications. When the components of the SPPB were considered separately systolic blood pressure 110-129 mmHg was associated with worse chair stand when compared with 130-159 mmHg. Although both of the higher blood pressure categories were associated with higher SPPB score, a systolic blood pressure of ≥160 mmHg was not associated with a substantial improvement in SPPB score when compared to 130-159 mmHg. This finding suggests that raising the systolic blood pressure target as recently recommended by the JNC 8 for older individuals could optimize physical performance without the adverse events that are associated with systolic blood pressures greater than 160 mmHg among patients on dialysis [5]. It was particularly notable in our study that 56% of patients with systolic blood pressure <110 mmHg and 75% of those with systolic blood pressure 110-129 mmHg were prescribed at least one blood pressure medication.

Prior studies in non-dialysis populations have shown that strict blood pressure control may not improve survival beyond conventional control. The ACCORD trial revealed that strict blood pressure control in patients with diabetes was not associated with a statistically significant improvement in survival and led to more adverse events [1]. Among dialysis patients, observational studies have described a J-shaped phenomenon in which patients with predialysis blood pressures less than 120 mmHg had higher cardiovascular and all-cause mortality when compared with patients with higher blood pressure, raising the possibility that strict blood pressure control could have adverse health consequences [16]. However, these studies had not considered physical performance and had not controlled for use of antihypertensive medication. Although lower postdialysis blood pressure has also been associated with higher mortality [20], we limited our analysis to predialysis blood pressure because physical function testing was performed prior to hemodialysis. Fluid overload could have caused an increase in predialysis blood pressure and worse physical function scores, but these associations would bias the results to the null.

End-stage kidney disease may be a model of accelerated aging, with cardiovascular and muscle mass changes typically observed in the elderly occurring even among younger patients [21]. Recent studies, focusing on the concept of physiologic age, have stratified outcomes in elderly based on physical performance tests [4]. The physiologic similarities between elderly and hemodialysis patients and the need for higher blood pressures to maintain adequate systemic perfusion could explain the association between higher systolic blood pressure and better physical function that we observed. We determined that patients with blood pressure <110 mmHg did not have significantly worse physical function than the 110-129 mmHg reference group which suggests that both traditional targets and tight blood pressure control may be associated with worse physical performance than higher blood pressure levels in this patient population.

Neither history of heart failure nor lower hemoglobin was significantly associated with a higher odds of being in the lowest SPPB in multivariable analysis. The lack of association with heart failure could be because the SPPB is a brief test that emphasizes mobility and strength and is not a test of endurance. Alternatively, the lack of association could reflect the common tendency for dialysis patients with fluid overload to be given a diagnosis of heart failure even in the absence of a structural cardiac abnormality. The association between albumin and physical performance was expected as higher albumin is reflective of better nutritional status or less inflammation. The association between physical performance and diabetes mellitus has also been described previously [19]. When we examined the individual components of the SPPB, we found that gait speed and sit to stand were better among patients with higher blood pressure, but we did not find an association between the balance component of the SPPB and blood pressure. These findings are compatible with the hypothesis that dynamic balance and function may be more susceptible to the effects of blood pressure than static balance.

To put the SPPB scores in context, the SPPB score has been predictive of mortality and nursing home admissions in elderly populations. ACTIVE/ADIPOSE participants had a lower percentage of participants scoring in the highest category (10-12) and a higher percentage scoring in the lowest category when compared with the 70-year old Established Populations for Epidemiologic Studies of the Elderly (EPESE) cohort. [13] SPPB scores in our participants can be compared to those of participants in the Frequent Hemodialysis Network (FHN) trial. The mean SPPB score in our cohort (7.8, SD 3.9) was lower than among FHN participants (8.5) who may have been a healthier, more selected population. Indeed, participants in our study were older (57.3 vs. 50.7 years). Older age, African-American race, diabetes mellitus, and peripheral arterial disease were all associated with lower SPPB scores among FHN participants, similar to our findings [19]. However, the association between systolic predialysis blood pressure and physical function was not addressed in the FHN.

Strengths of our study include careful direct measurement of physical performance and use of the SPPB, which allows comparison with other dialysis and non-dialysis populations. Information about prescription medication was carefully collected and available for analysis. Nevertheless, several limitations of our study should be addressed. First, we relied on blood pressure data that were obtained in clinical practice rather than under carefully controlled conditions. Although this could introduce some additional variation, such variation would tend to bias our results to the null. In addition, our data mirror the information that physicians have available for clinical decision-making. Second, the cross-sectional analyses precluded us from determining whether low blood pressure itself led to poor physical performance as we hypothesized or if patients with poor physical performance had low blood pressures due to unmeasured comorbidities. Third, we did not have information on whether patients were taking antihypertensive medications as prescribed or the indication for the prescribed medication. Fourth, we relied on diagnostic codes for heart failure, without capturing detailed echocardiographic data on cardiac function, so we could not determine whether differences in cardiac function might have contributed to the observed results.

## Conclusion

Our findings demonstrate an association between lower blood pressure and poorer physical performance among a cohort of patients on hemodialysis. The association was present regardless of age or antihypertensive therapy. Although management decisions cannot be based upon these data alone, these findings raise the possibility that aggressive blood pressure control in patients on hemodialysis could lead to adverse consequences, and the risk-benefit tradeoff of aggressive blood pressure control, particularly in low-functioning hemodialysis patients, should be reexamined. In addition, future randomized trials of blood pressure control among hemodialysis patients should include physical performance measures.

## Abbreviations

ACCORD: Action to control cardiovascular risk in diabetes
ESRD: End-stage renal disease
SPPB: Short physical performance battery
USRDS: United States renal data system
ACTIVE/ADIPOSE: A cohort study to investigate the value of exercise/analyses designed to investigate the paradox of obesity and survival in ESRD
EPESE: Established populations for epidemiologic studies of the elderly
FHN: Frequent hemodialysis network.
